# RapTB: a lung-derived hemoglobin fragment with activity against *Mycobacterium tuberculosis*

**DOI:** 10.3389/fmicb.2025.1669022

**Published:** 2025-10-24

**Authors:** Leonard Klevesath, Reiner Noschka, Thomas Vomhof, Jacky Mohnani, Mark Grieshober, Jens Michaelis, Paul Walther, Armando Rodriguez, Nico Preising, Clarissa Read, Sebastian Wiese, Ludger Ständker, Dietmar R. Thal, Jan Münch, Steffen Stenger

**Affiliations:** ^1^Ulm University Medical Center, Ulm, Germany; ^2^Institut of Medical Microbiology and Hygiene, Medical Facility, Ulm University, Ulm, Germany; ^3^Institute of Biophysics, Ulm University, Ulm, Germany; ^4^University of Ulm, Ulm, Germany; ^5^Central Facility for Electron Microscopy, Ulm University, Ulm, Germany; ^6^Core Unit Mass Spectrometry and Proteomics, Ulm, Germany; ^7^Core Facility of Functional Peptidomics, Ulm University, Ulm, Germany; ^8^Laboratory of Neuropathology, Department of Imaging and Pathology, Leuven Brain Institute, KU- Leuven, Leuven, Belgium; ^9^Institut of Molecular Virology, Ulm University, Ulm, Germany

**Keywords:** tuberculosis, antimicrobial peptides, human, lung, endogenous peptide

## Abstract

Tuberculosis (TB) remains difficult to treat due to the need for prolonged multidrug therapy and the global rise of drug-resistant *Mycobacterium tuberculosis* (*Mtb*) strains. Endogenous antimicrobial peptides (AMPs) have emerged as promising candidates for host-directed therapies. Given the pulmonary nature of TB, we hypothesized that human lung tissue contains peptides with intrinsic antimycobacterial activity. We screened a peptide library derived from human lung tissue and identified a 39-amino-acid C-terminal fragment of β-hemoglobin (HBB(112–147)), referred to as RapTB, with potent activity against *Mtb*. Recombinant RapTB exhibited dose-dependent inhibition of extracellular *Mtb*, reaching ~60% activity at 50 μM. Electron microscopy revealed mycobacterial cell wall disruption as a likely mechanism. RapTB was non-toxic to primary human macrophages and efficiently internalized by *Mtb*-infected cells. However, it did not co-localize with intracellular bacilli and failed to limit intracellular replication. HBB-derived fragments such as RapTB have previously been identified in human tissues and are known to exhibit broad-spectrum antimicrobial activity. Our findings extend this functional class to include antimycobacterial activity and suggest a potential role for RapTB in the early, extracellular phase of host defense against TB.

## 1 Introduction

More than 125 years after its discovery by Robert Koch, *Mtb* remains one of the most devastating pathogens worldwide, causing over 10 million new infections and 1.5 million deaths annually (World Health Organization, 2023). Despite the availability of effective antibiotics, the standard treatment requires a prolonged combination therapy of at least 6 months with two to four drugs. This regimen is often associated with severe side effects and poor patient compliance. The alarming rise of multidrug-resistant and extensively drug-resistant strains further hampers therapeutic success (Khawbung et al., 2021). Given these challenges, there is an urgent need for novel, safe, and effective therapeutic strategies that can complement or enhance current anti-tuberculosis regimens. Antimicrobial peptides (AMPs) have emerged as promising candidates in this context. AMPs are short, mostly cationic peptides that typically act by disrupting microbial membranes, thereby exhibiting broad-spectrum activity against bacteria, fungi, and viruses (Yeaman and Yount, 2003). They are evolutionarily conserved components of innate immunity and have been isolated from diverse sources including bacteria, plants, invertebrates, and mammals.

In humans, a rich diversity of bioactive peptides—collectively termed the peptidome—can be found in tissues and body fluids. The peptidome encompasses not only classical proteolytic fragments of larger precursor proteins but also peptides derived from short open reading frames, non-canonical translation events, alternative splice variants, and small secreted proteins. Despite its complexity and biological relevance, the human peptidome remains largely unexplored as a reservoir of endogenous antimicrobial compounds. Endogenous AMPs offer several advantages over conventional antibiotics. As naturally occurring host molecules, they are less likely to induce adverse side effects or trigger immune reactions. Their mechanism of action, typically based on electrostatic interactions with bacterial membranes, makes the development of resistance less likely. Furthermore, their relatively small size facilitates large-scale chemical synthesis at reasonable cost (Hancock and Sahl, 2006; Yeaman and Yount, 2003). To systematically explore the human peptidome for novel AMPs, we previously established a platform to generate peptide libraries from various human tissues and fluids and to screen these libraries for antimicrobial activity (Münch et al., 2014; Bosso et al., 2018). Using this approach, we previously identified the antimycobacterial peptides Angie1 and Lys-H1 from serum (Noschka et al., 2021; Maier et al., 2024) and NapFab from bronchoalveolar lavage fluid (Beitzinger et al., 2021). Since tuberculosis is primarily a pulmonary disease, and more than 95% of infected individuals are able to contain the pathogen without ever developing active disease (Migliori et al., 2021), we hypothesized that the human lung harbors innate defense molecules capable of controlling *Mtb* infection. To test this, we generated a peptide library from healthy human lung tissue, screened it for activity against virulent *Mtb* and functionally characterized the most potent bioactive molecule (HBB(112–147, RapTB).

## 2 Materials and methods

### 2.1 Generation of a peptide library from human lung tissues

A sample of 6,500 g of lung from deceased patients was subjected to homogenization. Peptide/protein extraction was performed by acidifying the sample with acetic acid to pH 3, followed by centrifugation at 16000 rpm for 15 min, and filtration (20, 8, 5, 3, 1.2, 0.45 μm) of the supernatant. Further, the filtered lung extract was subjected to ultrafiltration (cut off: 30 kDa), yielding a sample enriched in peptides and small proteins. The chromatographic fractionation of the ultrafiltrate sample was performed by using a reversed-phase (PS/DVB) HPLC column Sepax Poly RP300 (Sepax Technologies, Newark DE, USA 260,300–30,025) of dimensions 5 × 25 cm (10 μm), at a flow rate of 100 ml/min with the gradient program (min/%B): 5/5,30/50,50/100,55/5, being A, 0.1% TFA (Merck, 1,082,621,000) in ultrapure water, and B,0.1% TFA in acetonitrile (J. T. Baker, JT9012-3). Fifty-five reversed-phase chromatographic fractions of 100 ml were collected to constitute the lung peptide bank, from which 1 ml-aliquots (1%) were lyophilized and used for antimicrobial activity testing. For further purification of the active fractions, a Luna reversed phase C18 HPLC column (Phenomenex, USA) of dimensions 2.1 × 25 cm (5 μm) was used at a flow rate of 12.5 ml/min, with the gradient program (min/%B): 0/5, 5/5, 27/25, 47/50, 68/100, 75/5. Next, an Aeris TM widepore XB-C18 reversed-phase HPLC column (Phenomenex, USA) of dimensions 4.6 × 25 cm (3.6 μm) was used at a flow rate of 0.8 ml/min, with the gradient program (min/%B):0/5, 5/20, 50/45. The solvents used for both separations were A, 0.1% Trifluoroacetic acid (TFA) (Merck,1,082,621,000) in ultrapure water, and B, 0.1% TFA in acetonitrile (J. T. Baker, JT9012-3).

### 2.2 Evaluation of peptide fractions for activity against Mtb

To assess mycobacterial viability, we measured the incorporation of the radioactively labeled 5.6-^3^H-uracil (ART-0282, Biotrend, Cologne, Germany) into bacterial RNA (Noschka et al., 2021). Sonicated Mycobacteria (2 × 10^6^) were incubated with the peptides in Middlebrook 7H9 broth in a 96-well plate. Each condition was performed in triplicate, with rifampin (2 μg/ml, for *Mtb*) used as controls. After 72 h ^3^H-uracil (0.3 μCi/well) was added, and the cultures were incubated for an additional 18 h. Following incubation, the mycobacteria were inactivated by treatment with 4% paraformaldehyde (PFA, Sigma-Aldrich) for 30 min. The samples were then transferred onto glass fiber filters (Printed Filtermat A, PerkinElmer) using a Filtermat Harvester (Inotech). The filters were dried in a microwave at 240 W for 5 min and sealed with a layer of solid scintillant wax (MeltiLex, PerkinElmer) at 75 °C. Radioactivity was measured using a β-counter (Sense Beta, Hidex). Antimicrobial activity was calculated by dividing the counts per minute (cpm) of the treated sample by the cpm of the untreated sample and multiplying by 100.

### 2.3 Mass spectrometry identification of active molecules

Identification of active peptides in active fractions was performed on a nano LC-Orbitrap Elite Hybrid mass spectrometry system (Thermo Fisher Scientific, Bremen, Germany). The samples were reduced with 5 mM Dithiothreitol (DTT) for 20 min at RT, carbamidomethylated with 50 mM iodoacetamide for 20 min at 37 °C and quenched with 10 mM DTT. A 15 μl-aliquot was analyzed as previously described (Rodríguez-Alfonso et al., 2022). Database searches were performed using PEAKsXPro (PEAKs studio 10.6) (Zhang et al., 2012). For peptide identification, MS/MS spectra were correlated with the UniProt human reference proteome (19). Carbamidomethylated cysteine was considered as a fixed modification along with oxidation (M) and deamidation (NQ) as variable modifications. False discovery rates were set on the peptide level to 1%.

### 2.4 Peptide origin and synthesis

RapTB was extracted and purified from human lung lysate. The peptide RapTB (VCVLAHHFGKEFTPPVQAAYQKVVAGVANALAHKYH) was synthesized by PSL Heidelberg (Heidelberg, Germany) using Fmoc chemistry (Noschka et al., 2021). The peptides were purified to >95% purity using reverse-phase HPLC. To ensure valid comparisons between experiments, all concentrations were expressed in molarity.

### 2.5 Source and culture of mycobacteria

Mycobacterial strains used in this study are listed in Table 1. Mycobacteria were cultured, stored, and amplified as previously described (Noschka et al., 2021). Representative vials were thawed, and the viable colony-forming units (CFU) were enumerated on Middlebrook 7H11 agar plates (BD Biosciences). Live-dead staining (BacLight, Invitrogen) of bacterial suspensions using fluorochrome substrates indicated that the bacterial viability was greater than 90%. Before use, aliquots were sonicated in a water bath for 10 min at 40 kHz and 110 W at room temperature to disrupt small bacterial aggregates. Mycobacterial cultures were grown on Middlebrook 7H11 agar plates, with 2 × 10^6^ sonicated mycobacteria spread on the plates in logarithmic dilutions (1:10, 1:100, and 1:1000). The plates were then incubated for 14 days at 37 °C. Following incubation, the colonies were counted to determine CFU.

**Table 1:** Mycobacterial strains.

Name	Source
*Mycobacterium tuberculosis*	ATCC 27294^a^
GFP-positive *Mycobacterium tuberculosis*	H37Rv-GFP

a ATCC (American Type Culture Collection) Manassas, VA, USA.

### 2.6 Generation of macrophages and infection with Mtb

Human peripheral blood mononuclear cells (PBMCs) were isolated from buffy coats of anonymous donors (Institute of Transfusion Medicine, Ulm University) using density gradient centrifugation (Ficoll-Paque Plus, GE Healthcare). Monocytes were then selected based on their ability to adhere to plastic and were thoroughly washed. To generate monocyte-derived macrophages, the cells were cultured in M-SFM supplemented with granulocyte–macrophage colony-stimulating factor (GM-CSF, 10 ng/ml, Miltenyi) for 6 days as previously outlined (Noschka et al., 2021). After this, macrophages were detached using 1 mM Ethylene diamine tetraacetic acid (EDTA) (Sigma-Aldrich) and subsequently infected in 6-well plates with single-cell suspensions of *Mtb* at a multiplicity of infection (MOI) of 5. After a 2-h incubation, the cells were thoroughly washed to remove extracellular bacteria and harvested using 1 mM EDTA (Sigma-Aldrich).

### 2.7 Scanning Electron microscopy

Initially, 5 × 10^6^ *Mtb* were seeded into a 24-well plate containing Middlebrook 7H9 broth and incubated with 50 μM RapTB for 72 h. Following incubation, the bacteria were harvested, transferred to screw-cap tubes, and centrifuged at 10,000 rpm for 10 min. The supernatant was discarded, and the bacterial pellet was resuspended and fixed in 100 μl of 4% paraformaldehyde (PFA) for 20 min. Subsequently, chemical fixation was performed using 2.5% glutaraldehyde (prepared in phosphate-buffered saline (PBS) with 1% sucrose) for 1 h. The bacteria were then post-fixed with 2% osmium tetroxide (OsO₄) in PBS for 1 h at room temperature. Dehydration was carried out through a graded series of propanol concentrations (30, 50, 70, 90, and 100%), with each step lasting 5 min (Walther et al., 2010). Samples were then subjected to critical point drying using carbon dioxide as the transitional medium (Critical Point Dryer CPD 030, Bal-Tec, Liechtenstein). The dried samples were rotary coated in a BAF 300 freeze-etching device (Bal-Tec) using electron beam evaporation with a 3 nm platinum–carbon layer applied at a 45° angle. Imaging was performed using a Hitachi S-5200 in-lens field-emission scanning electron microscope (Hitachi High-Tech, Tokyo, Japan) at an accelerating voltage of 10 kV, using the secondary electron signal.

### 2.8 Assessment of macrophage viability

For *in vitro* analysis, 1 × 10^5^ macrophages were seeded in a 96-well plate and incubated with RapTB for 24 h. Subsequently, 10% PrestoBlue reagent (Thermo Fisher, Waltham, MA, USA) was added to each well and incubated for 20 min. The conversion of the non-fluorescent, resazurin-based substrate to fluorescent resorufin by mitochondrial enzymes of metabolically active cells was used to assess cytotoxicity, as previously described (Noschka et al., 2021).

### 2.9 Uptake of RapTB by macrophages-confocal microscopy

*Mtb*-infected macrophages were seeded onto 8-chamber slides (Thermo Fisher Scientific) with a final volume of 100 μl which is equivalent to 100,000 macrophages. Following cell fixation with paraformaldehyde (PFA, Sigma; final concentration 4%), non-specific binding sites were blocked using a blocking buffer for 1 h at room temperature. This step also permeabilized the cell wall for intracellular staining. Permeabilization was followed by incubation with primary antibodies against RapTB (1:250) or MHC class II (1:300) for 1 h. Cells were then incubated with secondary antibodies: Cy2-conjugated donkey anti-rabbit (1:200), for another hr. at room temperature. Nuclei were counterstained with 1 μg/ml DAPI for 10 min, and slides were mounted using Aquatex (Merck, Darmstadt, Germany). Images were acquired using a Zeiss LSM 710 inverted laser scanning confocal microscope (Zeiss, Oberkochen, Germany) and analyzed with ImageJ software (version 1.53c). All antibodies used are listed in Table 2.

**Table 2:** Antibodies.

Antibody	Source
Donkey anti Rabbit Cy2	Dianova
Diamidinophenylindole (DAPI)	Sigma
Anti-Human HLA (Human Leukozyten-Antigen)-DR	Leinco Technologies
LI-COR secondary antibody IRDye 800CW Goat α Rabbit	LI-COR Corporate Office
Goat α Mouse Cy5	Dianova
Anti-Hemoglobin-Beta antibody	Abcam

### 2.10 Single molecule localization microscopy and colocalization analysis of RapTB with Mtb

Primary human macrophages were seeded in 35 mm ibidi-dishes with glass bottom (Ibidi GmbH, 81,158), infected with GFP-positive *Mtb* (Table 1) at an MOI of 10 and incubated with 500 nM RapTB. The cells were fixed with 4% paraformaldehyde solution (15,714, Electron Microscopy Sciences, Hatfield, USA) before being incubated with blocking buffer (1% bovine serum albumin (BSA) and 0.1% Triton X-100 in PBS) for 2 h. Anti-Hemoglobin subunit beta antibody [EPR20614] (Abcam, Cambridge, UK, ab214049) at a dilution of 1:200 in blocking buffer was added overnight at 4 °C. After three washing steps with blocking buffer the secondary antibody Goat anti-Rabbit IgG (H + L) Cross-Adsorbed Secondary Antibody, Alexa Fluor 647 (Thermo Fisher, Waltham, MA, USA) was diluted 1:200 in blocking buffer and added for 1 h at room temperature, followed by another three washing steps. Prior to imaging, the buffer is replaced with degassed dSTORM imaging buffer at pH 7.4 containing 100 mM β-Mercaptoethylamine (30070), 0.02 mg/ml catalase (C30), 0.5 mg/ml glucose oxidase (49180) and 200 mM D-(+)-glucose (G7528) in PBS (all Sigma Aldrich). dSTORM imaging was performed on a custom-built microscope optimized for single molecule localization microscopy first described here (Schoen et al., 2016). The Alexa Fluor 647 fluorophores attached to RapTB were excited with a 638 nm Omicron LuxX 638–300, 200 mW laser (Omicron Laserage Laserprodukte GmbH, Germany), the GFP expressed by *Mtb* were excited with a 488 nm iBEAM-SMART-488-S, 60 W laser (TOPTICA Photonics AG, Germany) and photoactivation was done with a 405 nm iBEAM-SMART-405-S, 120 mW laser (TOPTICA Photonics AG, Germany). Imaging was performed in HILO mode to optimize single molecule SNR. The 638 nm channel was imaged onto an iXon 897 Ultra EMCCD camera and the 488 nm channel was imaged onto an iXon 897 EMCCD camera (both Andor Technology, Oxford Instruments, Belfast, UK) with an image pixel size of 133 nm and an exposure time of 40 ms. Before starting the dSTORM measurement the sample was excited by the 638 nm laser at maximum laser power to drive a majority of fluorophores into the transient dark state. The dSTORM raw data consists of 10.000 frames. Post processing was performed using the SMAP SMLM platform (Ries, 2020). The localizations were filtered for localization precision between 10 and 50 nm and a PSF width of 110-200 nm. Drift correction was performed based on sub-reconstruction cross-correlation. Localization density quantification was done by comparing the number of RapTB molecules per area inside the diffraction-limited regions of the *Mtb* to the RapTB density in the cytosol of the respective cells in the absence of *Mtb*. dSTORM imaging was performed on macrophages from three different donors.

### 2.11 Quantification of intracellular mycobacterial growth: mycobacterial growth inhibition assay (MGIA)

Macrophages were infected with *Mtb* (MOI 5, 2 h) and extracellular bacilli were removed by extensive washing. The cells were then harvested and seeded into a 24-well plate at a density of 1 × 10^5^ cells in 300 μl M-SFM. Infected macrophages were treated as specified, and after 72 h., cells were lysed by adding 200 μl of water. The resulting lysates were transferred to mycobacteria growth indicator tubes (MGIT, BD Biosciences, Franklin Lakes, USA) and supplemented with 800 μl of a supplement containing oleic acid, albumin, dextrose and catalase (Ölsäure-Albumin-Dextrose-Katalase (OADC), BD Biosciences). The tubes were incubated in a Bactec MGIT 320 system (BD Biosciences), which detects oxygen consumption and provides the exact time (in minutes) from the start of culture to the detection of bacterial activity (time to positivity). The number of viable bacilli was determined by comparing the time to positivity in the sample to a standard curve generated from the growth of tenfold serially diluted mycobacterial suspensions (10^3^ to10^7^ bacilli, in duplicate).

### 2.12 Statistical analysis

All statistical analyses described in the figure legends were conducted using GraphPad Prism v10 (GraphPad Software). Significance was determined using non-parametric tests for paired samples, including the Wilcoxon rank-sum test and paired t-test. A *p*-value of less than 0.05 was considered statistically significant.

## 3 Results

**Figure 1:**
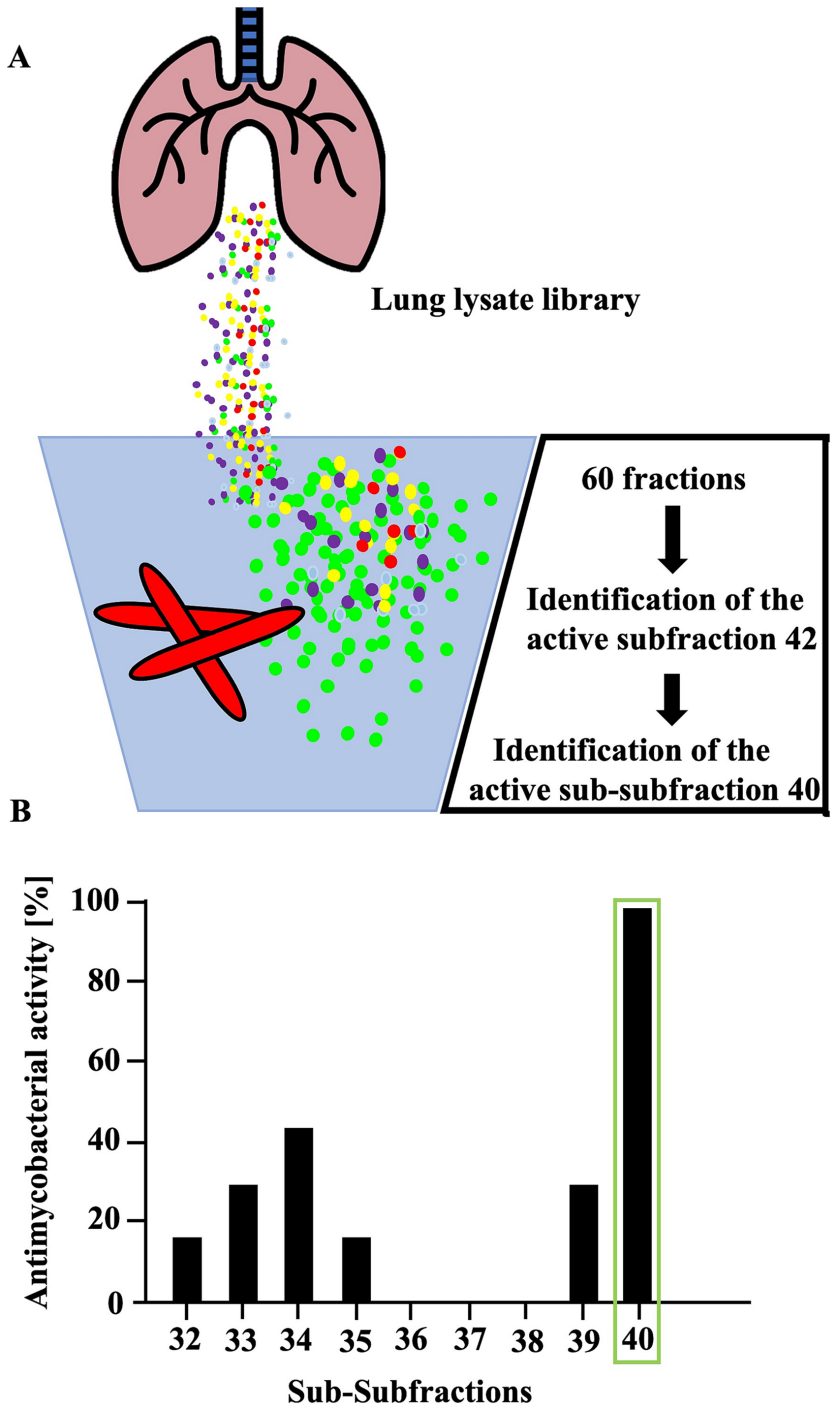
Activity of fractions obtained from human lung against extracellular *Mtb*. **(A)** To identify active peptides, lung lysate was repeatedly fractionated and assessed for activity against *Mtb*. **(B)** A total of sixty individual fractions from a lung lysate library were tested for antimicrobial activity against extracellular *M. tuberculosis*. Fractions exhibiting more than 30% inhibitory activity were further subfractionated using high-performance liquid chromatography (HPLC). Only fractions 32 to 40 are shown in the figure. Fractions were incubated with bacteria for 96 h; during the final 24 h, ^3^H-uracil was added to monitor bacterial RNA synthesis. Uptake of ^3^H-uracil was quantified by scintillation counting with a β-counter. Antimicrobial activity was determined by comparing radioactivity levels to those of an untreated control. Experiments were performed in triplicates. Mass spectrometric analysis of subfraction 40 identified a peptide corresponding to amino acids 112–147, a fragment of hemoglobin B.

**Figure 2:**
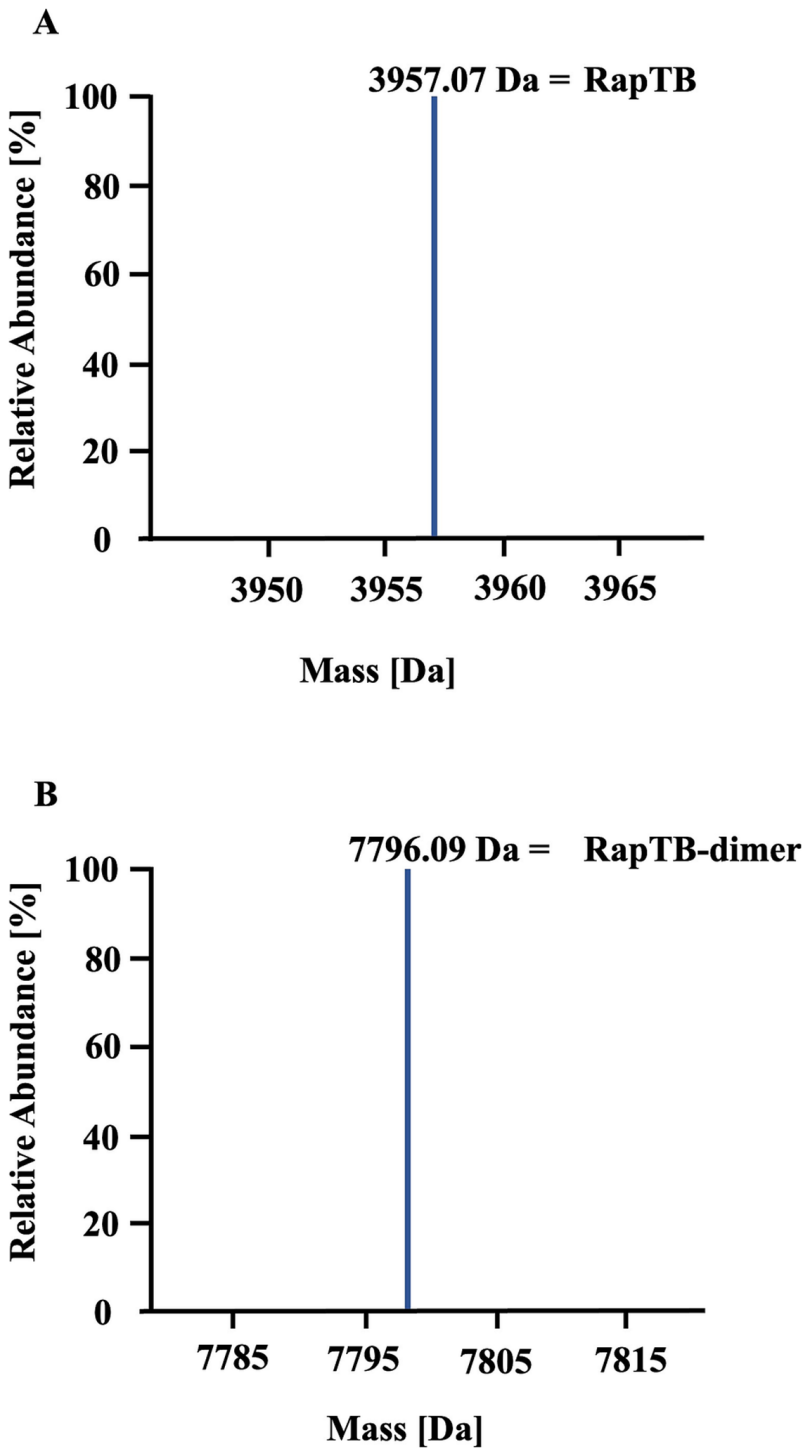
Identification of RapTB as the active component in subfraction 40. Analysis of subfraction 40 by mass spectrometry (nanoLC-Orbitrap Elite system). **(A)** Deconvoluted mass spectrum of the unmodified sample. **(B)** Deconvoluted mass spectrum of the sample after carbamidomethylation.

To identify antimicrobial peptides from lung lysate, 60 fractions were screened for antimetabolic activity against *Mtb* (Figure 1). Fraction 42 exhibited activity exceeding 30%. Repeated sub-fractionation led to the identification of sub-subfraction 40, which showed antimetabolic activity of over 90%. Mass spectrometry analysis of subfraction 40 revealed a signal at 7798.09 Da (deconvoluted spectrum), which turned into a signal at 3957.07 Da after carbamidomethylation, indicating the presence of a dimer formed by one intermolecular disulfide bridge (Figure 2). The MS/MS sequencing of the carbamidomethylated sample revealed the presence of HBB 112–147, VCVLAHHFGKEFTPPVQAAYQKVVAGVANALAHKYH (Supplementary Figure 1). This C-terminal peptide has previously been detected in human placenta and menstrual blood and showed antimicrobial activity, extracellular bacteria and viruses (Liepke et al., 2003; Mak et al., 2004; Mak et al., 2007; Groß et al., 2020).

**Figure 3:**
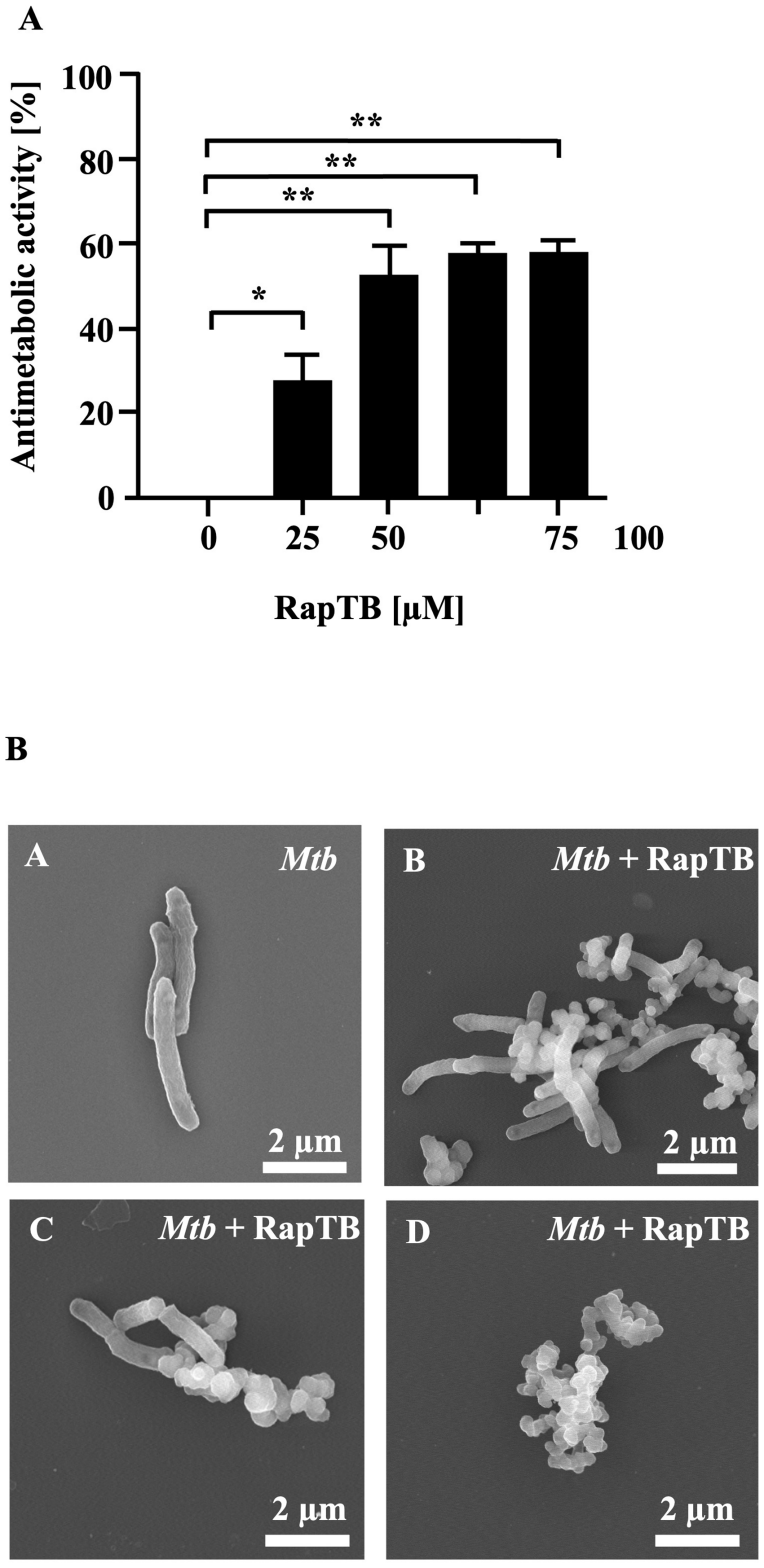
Effect of RapTB against extracellular *Mtb*. **(A)** 2 × 10^6^ extracellular *Mtb* were incubated with increasing concentrations of RapTB for 3 days, followed by an additional 16-h incubation with ^3^H-uracil. Radioactive uptake was measured by scintillation counting. Antibacterial activity was calculated by comparing the counts per minute (cpm) of treated samples to those of the untreated control. All conditions were tested in triplicate. The graph shows a representative result of *n* = 6 independent experiments. **(B)** Extracellular *Mtb* was either treated with RapTB or left untreated for 3 days before being processed for scanning electron microscopy. Representative images depict untreated **(A)** and RapTB-treated *Mtb* **(B–D)**. Imaging was performed using a Hitachi S-5200 scanning electron microscope at magnifications ranging from 40,000 × to 50,000×. Mycobacteria from three independent experiments were analyzed. Representative images are shown.

To validate the antitubercular activity of RapTB, the synthetic peptide was tested against extracellular *Mtb* in an antimetabolic assay. RapTB exhibited a maximum of 53% inhibition at 50 μM (Figure 3A). Higher concentrations did not further enhance activity. To investigate structural effects on the pathogen, *Mtb* was incubated with RapTB (50 μM) and analyzed by transmission electron microscopy. Compared to untreated bacilli, which appeared as intact rods, RapTB-treated *Mtb* displayed profound morphological alterations, including vesicle formation, membrane swelling, and loss of membrane integrity (Figure 3B), suggesting membrane disruption as a likely mode of action.

**Figure 4:**
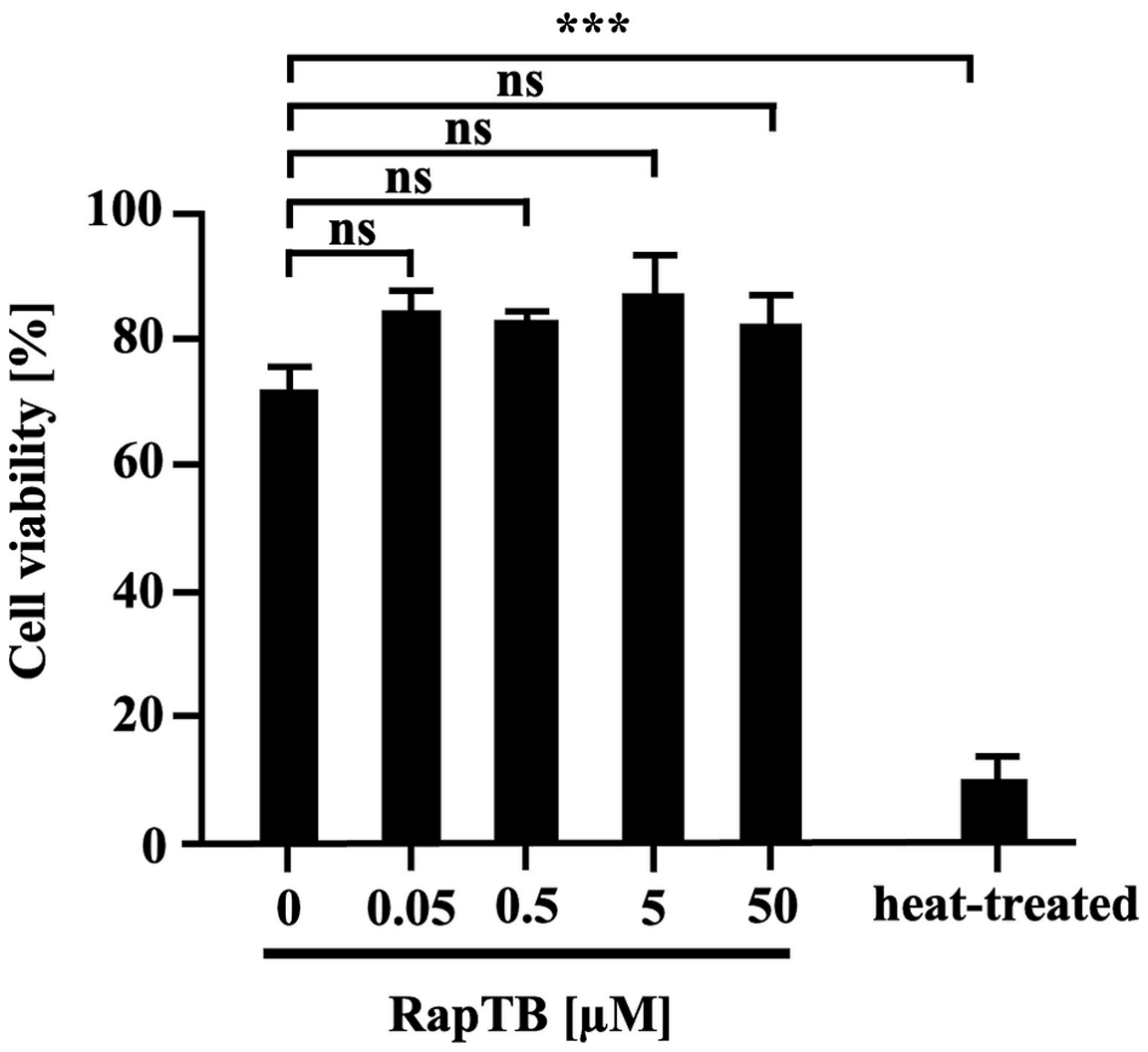
Effect of RapTB on the viability of human macrophages. 0.1 × 10^6^ macrophages were incubated with RapTB for 24 h. Cell viability was assessed using the PrestoBlue assay. Bar graphs represent the mean viability (%) ± SEM from six independent experiments.

Since *Mtb* primarily resides in macrophages, the cytotoxicity of RapTB was assessed against primary human macrophages after 24 h of exposure. At 50 μM, RapTB showed minimal cytotoxicity (13%). At lower concentrations (0.0005–5 μM), cell viability remained above 79% (Figure 4), indicating that RapTB is well tolerated by host cells.

**Figure 5:**
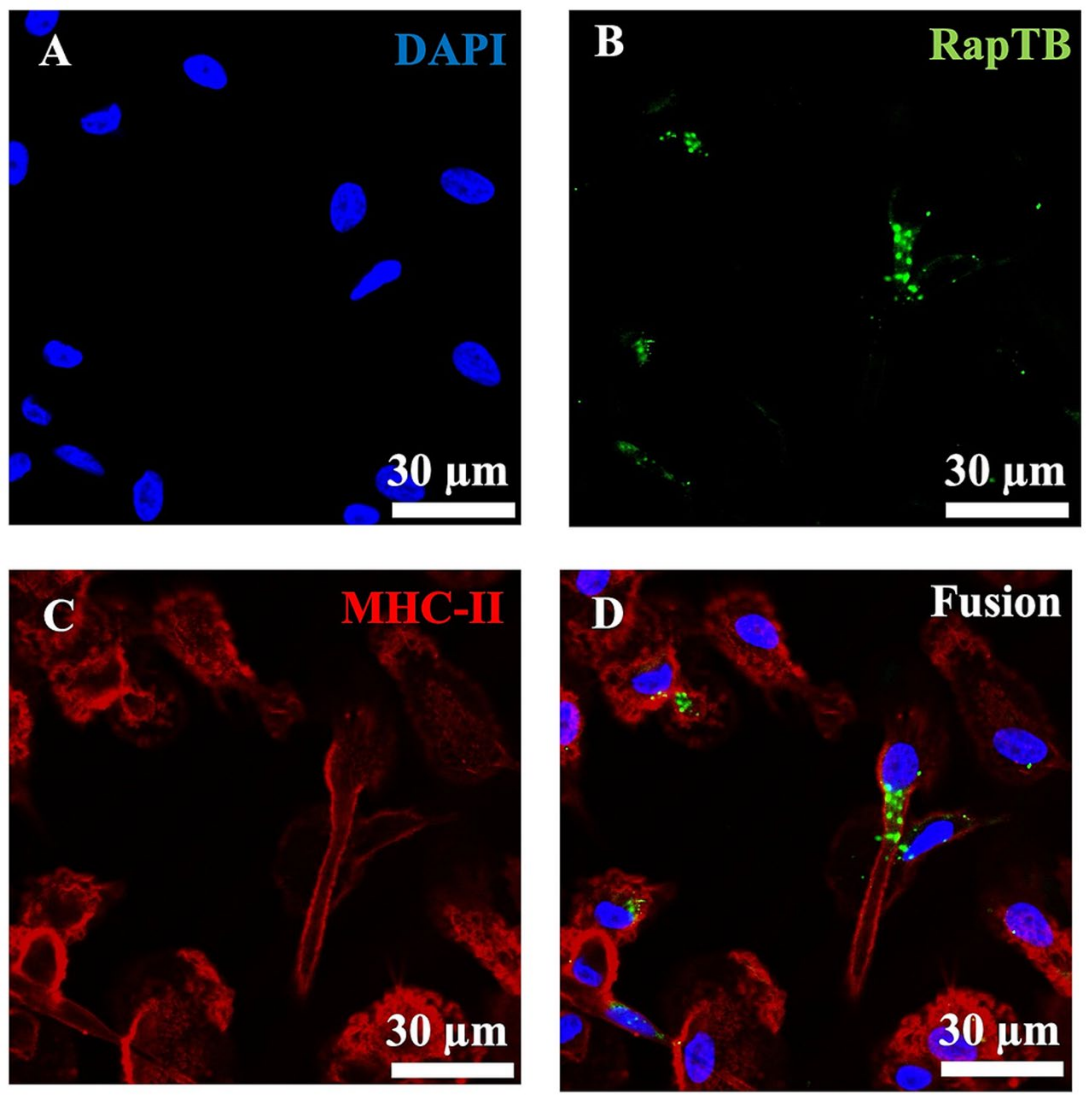
Uptake of RapTB by human macrophages. Confocal microscopy, Immunofluorescence staining of RapTB **i**n macrophages using an anti-HBB antibody and a Cy2-labelled secondary antibody. **(A)** Shows nuclear staining with DAPI, **(B)** shows RapTB staining, and **(C)** depicts MHC class II staining, a specific marker of macrophages. **(D)** Displays the merged overlay of all three channels. Original magnification ×63. Cells from three independent donors were analyzed. A representative image is shown.

To determine whether RapTB could reach intracellular *Mtb*, we investigated its uptake by macrophages using confocal laser scanning microscopy. Since a RapTB -specific antibody was required for detection in confocal microscopy and RapTB is a fragment of hemoglobin ß, a panel of commercially available anti-hemoglobin ß antibodies were tested for cross-reactivity, one of which recognized RapTB (Supplementary Figure 2). After 24 h, RapTB localized predominantly to vesicular structures near the nucleus (Figure 5). However, not all macrophages internalized the peptide, and no direct colocalization with intracellular bacilli was observed or it could not be detected at the sensitivity level of the microscope.

**Figure 6:**
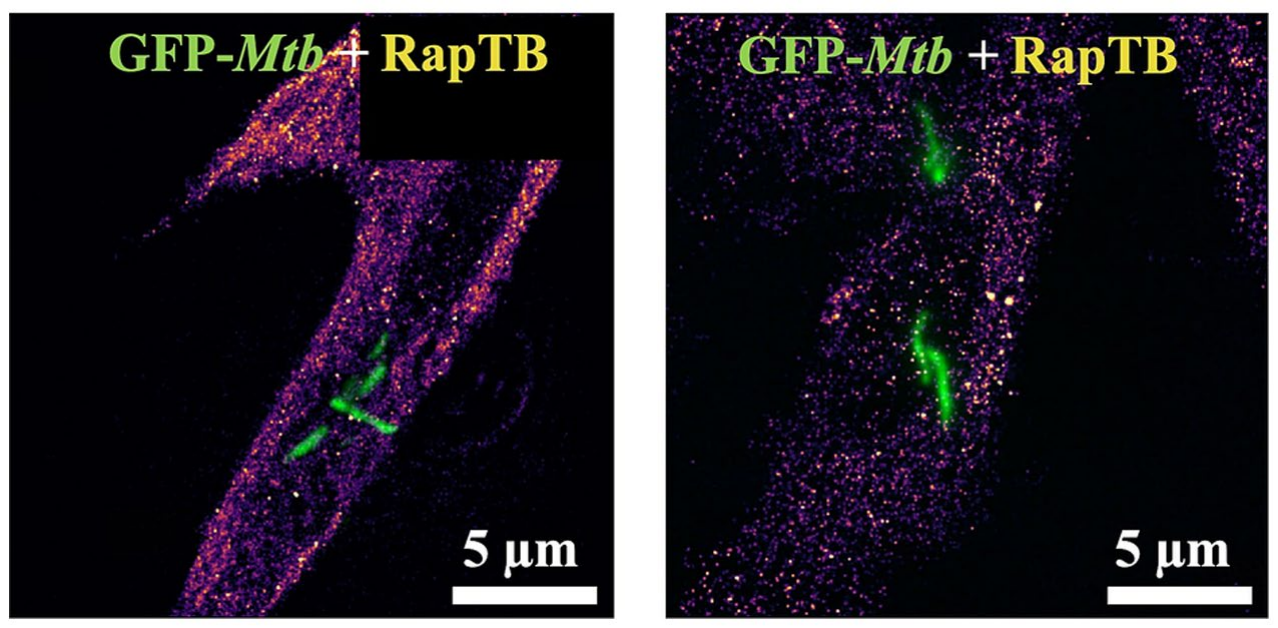
Uptake and localization of RapTB in *Mtb*-infected macrophages. Single molecule localization reconstruction (dSTORM) of RapTB in *Mtb*-infected primary human macrophages overlaid with the diffraction-limited images of *Mtb* expressing GFP (green). Cells from three independent donors were analyzed. Representative images are shown.

To investigate the RapTB localization on a molecular level in particular with respect to the intra-cellular *Mtb*, we used single molecule localization using dSTORM (Figure 6), resulting in super-resolution optical microscopy images. In the dSTORM-reconstructions of the macrophages treated with RapTB (0.5 μM), individual RapTB molecules could be localized throughout the cytosol of macrophages. The molecular density of RapTB inside the diffraction-limited region covered by the *Mtb* normalized by the cytosolic molecular density yields a value of 0.92±0.07 when averaged over all donors (*n* = 3) and reconstructions (*n* = 12). This demonstrates that RapTB does not preferentially colocalize with *Mtb* intracellularly. The fact that RapTB is underrepresented in the areas occupied by *Mtb* indicates, that *Mtb* reside in compartments with low peptide uptake.

**Figure 7:**
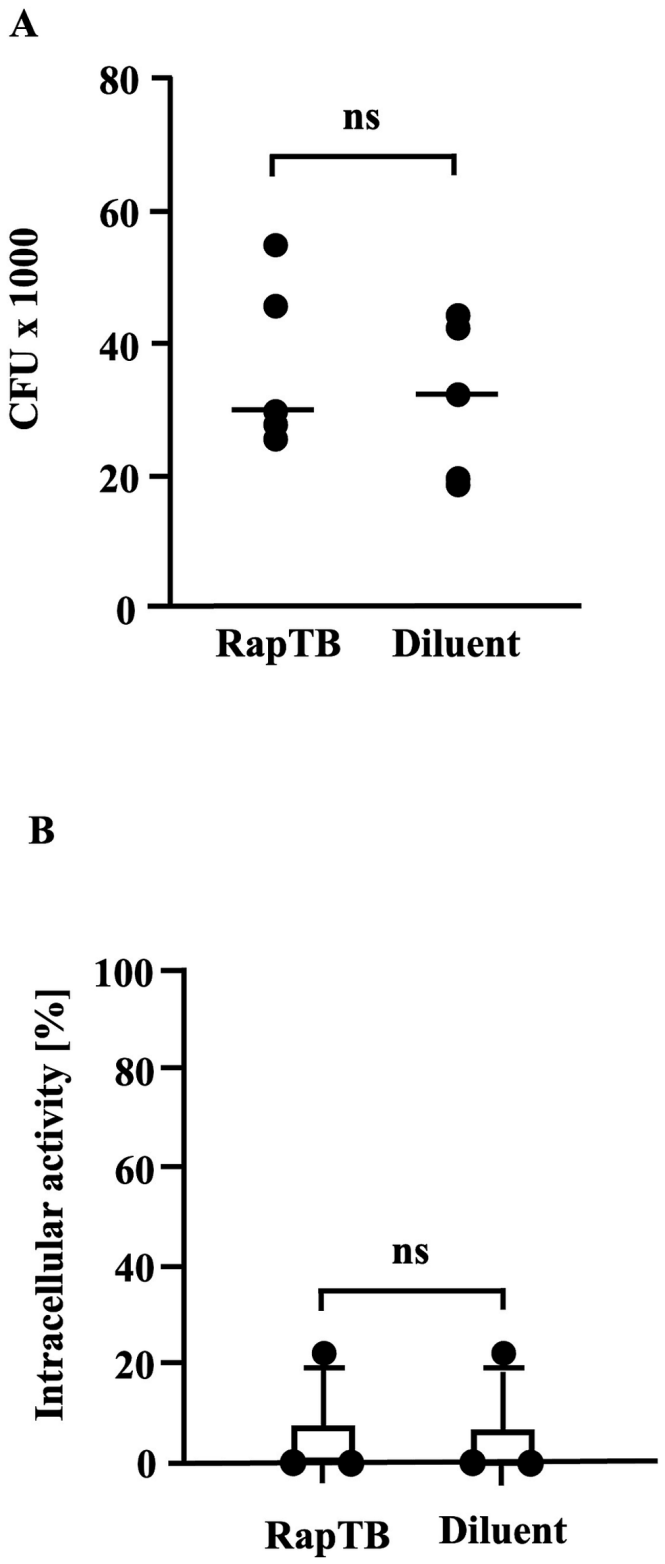
**(A)** Effect of RapTB on the viability of intracellular *Mtb*. Macrophages were infected with *Mtb* at a multiplicity of infection (MOI) of 5 and treated with RapTB for 4 days following the same protocol used for metabolic activity assays. Subsequently, the cells were lysed, and the released bacteria were plated onto 7H11 Middlebrook agar. After 14 days of incubation, colony-forming units (CFUs) were enumerated. The bar graph represents the Mycobacterial viability rates from five independent experiments (*n* = 5). **(B)** Effect of RapTB on the viability of intracellular *Mtb*. (MGIA). Macrophages were infected with *Mtb* and subsequently treated with RapTB. After 4 days, cells were lysed using distilled water and transferred to MGIT tubes. Time to positivity (TTP) was measured using the BACTEC system. The number of viable bacilli was estimated by comparing the TTP values to a standard curve generated with defined quantities of extracellular *Mtb*. As a positive control, the antimicrobial peptides Gran1 (1 μM) and NapFab/ DMSN (5 μM) were used (data not shown). The graph presents mean values ± SEM from three independent experiments using macrophages from different donors (*n* = 3).

To assess the antimycobacterial of RapTB, infected macrophages were treated with RapTB (50 μM) for 4 days, followed by lysis and quantification of viable bacteria by determining the number of colony forming units on agar plates. No reduction in intracellular *Mtb* was observed (Figure 7A). This result was corroborated using the mycobacterial growth inhibition assay (MGIA) as an independent objective approach (Figure 7B). Together, these findings demonstrate that while RapTB is highly active against extracellular *Mtb*, it lacks efficacy against intracellular bacilli, likely due to inefficient colocalization with the pathogen inside host cells.

## 4 Discussion

Here, we report the identification and characterization of HBB(112–147), a 39-amino-acid C-terminal fragment of β-hemoglobin, now termed RapTB. RapTB exhibited potent antimycobacterial activity against extracellular *Mtb*, likely via disruption of the bacterial cell wall. Although RapTB was efficiently internalized by human macrophages, it did not co-localize with intracellular bacilli and failed to inhibit intracellular *Mtb*.

RapTB belongs to a family of endogenous antimicrobial peptides, known as hemocidins, which are generated by proteolytic cleavage of hemoglobin. We and others described this and related fragments in placenta and menstrual blood and demonstrated broad-spectrum antimicrobial activity against Gram-positive and Gram-negative bacteria, fungi, and HSV-2 (Liepke et al., 2003; Mak et al., 2004; Mak et al., 2007; Groß et al., 2020). These peptides were shown to be active under acidic conditions, with their efficacy modulated by ionic strength and divalent cations such as Mg^2+^ and Ca^2+^, and to act synergistically with other endogenous AMPs including HBD-1, LL-37, and HNP-1 (Mak et al., 2007). In those studies, HBB(112–147) was identified through independent biochemical approaches and shown to be processed by physiological proteases such as Napsin A and Cathepsin D (Liepke et al., 2003; Beitzinger et al., 2021). However, antimycobacterial activity had not been previously reported. Our current study extends the known functional profile of this peptide by demonstrating that RapTB exhibits potent activity against *Mycobacterium tuberculosis*, the causative agent of tuberculosis. This new activity was discovered through the screening of a human lung-derived peptide library, emphasizing the potential role of hemoglobin-derived AMPs in pulmonary host defense. The identification of RapTB in lung tissue- the primary site of *Mtb* infection- highlights its likely physiological relevance and introduces a new layer of function to this well-characterized peptide. While RapTB effectively killed extracellular *Mtb*, it failed to co-localize with intracellular bacilli and showed no activity in infected macrophages. This emphasizes the importance of cellular trafficking and intracellular access for antimicrobial function, especially in the context of intracellular pathogens such as *Mtb*.

The systematic screening of peptide libraries derived from human tissues has proven to be an effective strategy for identifying endogenous AMPs. Using this approach, we previously discovered angiogenin from hemofiltrate, which showed both extracellular and intracellular anti-*Mtb* activity (Noschka et al., 2021). In contrast to angiogenin, RapTB lacks intracellular activity but is highly effective against extracellular *Mtb*. Nevertheless, RapTB could contribute to protection against tuberculosis during the early stages of infection when the pathogens logarithmically replicate in the alveolar space before adaptive immunity is initiated (Ryndak and Laal, 2019). RapTB shares important biochemical features with angiogenin (122 aa) including its cationic charge that promotes interaction with negatively charged mycobacterial membranes (Hancock and Sahl, 2006). With a length of 36 amino acids, RapTB is structurally intermediate yet functionally distinct. Interestingly, RapTB is generated by proteolytic cleavage of hemoglobin by Napsin A (Beitzinger et al., 2021), indicating potential crosstalk between host proteases and AMP production.

Our findings also support a broader concept: the proteolytic processing of abundant precursor proteins such as hemoglobin can generate multiple antimicrobial peptides with complementary or synergistic activity. Proteases like Napsin A and Cathepsin D, which are active in lung and placenta, cleave hemoglobin into bioactive fragments including RapTB and related hemocidins (Liepke et al., 2003; Beitzinger et al., 2021; The UniProt Consortium et al., 2023; Walther et al., 2010). These peptides have been shown to act synergistically with other AMPs such as HBD-1 and LL-37 (Mak et al., 2007), suggesting that localized proteolysis may shape a combinatorial AMP response. This mechanism may enhance antimicrobial efficacy, broaden pathogen coverage, and reduce the likelihood of resistance development.

The presence of hemocidins in lung tissue may reflect erythrocyte leakage due to mechanical disruption or inflammation. This is particularly relevant in tuberculosis, where capillary erosion and hemoptysis are common features (Wang et al., 2024). Such events could release hemoglobin into the lung parenchyma, allowing local proteases to generate bioactive peptides that contribute to the innate immune response.

Despite its lack of intracellular activity in its native form, RapTB may still contribute to host defense by limiting early extracellular *Mtb* replication. Since one of the primary goals of TB therapy is to control this initial bacterial expansion, AMPs like RapTB may serve as valuable adjuncts, especially in cases of multidrug-resistant tuberculosis. In addition to direct antimicrobial activity, hemoglobin-derived peptides have been shown to enhance cytokine release and macrophage activation, suggesting potential immunomodulatory functions as well (Roth et al., 1994). Furthermore, as RapTB is efficiently internalized by macrophages, it could potentially complement the activity of conventional antimycobacterial drugs. For instance, antimycobacterial agents may alter phagosomal integrity or cytosolic trafficking, thereby enabling RapTB to access intracellular *Mtb* and act synergistically.

To overcome the current limitations in intracellular activity, several strategies may be pursued. One approach is to improve intracellular delivery using nanocarrier systems. Dendritic mesoporous silica nanoparticles (DMSNs) have shown promise for enhancing peptide uptake, providing sustained release, and maintaining low cytotoxicity (Xu et al., 2019; Croissant et al., 2018; Amin et al., 2021; Du and Qiao, 2015). Similarly, liposomal encapsulation can improve intracellular delivery and efficacy of AMPs (Alzahrani et al., 2022). Targeted delivery via conjugation to toxins like C3bot from *Clostridium botulinum*, which selectively enters monocyte-derived cells, offer the possibility to optimize targeting to infected macrophages (Fellermann et al., 2022). Nanodiamonds are an emerging platform for AMP delivery, enabling enhanced intracellular accumulation, real-time imaging, and co-delivery of multiple therapeutic agents (Chow et al., 2011; Chen et al., 2009; Wu and Weil, 2022). Another strategy to improve RapTB function involves chemical modification of the peptide backbone. Substitution of L-amino acids with D-enantiomers can increase serum stability and reduce immunogenicity, as shown in VEGF and other peptides (Kreil, 1997; Garton et al., 2018; Uppalapati et al., 2016). Such modifications could prolong the half-life of RapTB and protect it from proteolytic degradation.

In summary, we identified RapTB as a hemoglobin-derived AMP with potent activity against extracellular *Mtb*. Our findings extend the known antimicrobial spectrum of hemocidins and support a role for protease-generated AMPs in pulmonary host defense. Ongoing studies aim to optimize the stability and intracellular trafficking of RapTB using rational peptide design and advanced delivery systems, with the goal of evaluating its potential for adjunctive therapy in severe or drug-resistant tuberculosis.

## Data Availability

The raw data supporting the conclusions of this article will be made available by the authors, without undue reservation.

## Supplementary material

SUPPLEMENTARY FIGURE 1 Detection of HBB 112–147. Fragmentation spectrum showing the presence of HBB 112–147, VCVLAHHFGKEFTPPVQAAYQKVVAGVANALAHKYH.

SUPPLEMENTARY FIGURE 2 Detection of RapTB with hemoglobin-ß antibody. After Immunofluorescence staining of RapTB using an anti-HBB antibody and a Cy2-labelled secondary antibody, fluorescent emission is measured. The figure shows a titration of RapTB: 50 μM **(A)**, 5 μM **(B)**, 0.5 μM **(C)** und 0.05 μM **(D)**.

## Glossary

AMP: Antimicrobial peptide
BSA: Bovine serum albumin
CFU: Colony forming units
CPM: Counts per minute
DAPI: Diamidin-phenylindol
DMSN: Dendritic mesoporous silica nanoparticles
DTT: Dithiothreitol
EDTA: Ethylene diamine tetraacetic acid
GFP: Green Fluorescent Protein
GM-CSF: Granulocyte-macrophage colony-stimulating factor
HBB: Hemoglobin subunit ß
HRS: Hours
HSV-2: Herpes simplex virus type 2
MGIA: Mycobacterial growth inhibition assay
MOI: Multiplicity of infection
*Mtb*: *Mycobacterium tuberculosis*
OADC: Ölsäure-Albumin-Dextrose-Katalase
OsO₄: Osmium tetroxide
PBMC: Peripheral blood mononuclear cell
PBS: Phosphate buffered saline
PFA: Paraformaldehyde
TB: Tuberculosis
TFA: Trifluoroacetic acid
VEGF: Vascular endothelial growth factor
