# Supplementary figures and images for: RapTB: a lung-derived hemoglobin fragment with activity against Mycobacterium tuberculosis
Source: Front Microbiol. 2025 Oct 24;16:1669022. doi: 10.3389/fmicb.2025.1669022 (PMC12592065; doi:10.3389/fmicb.2025.1669022)

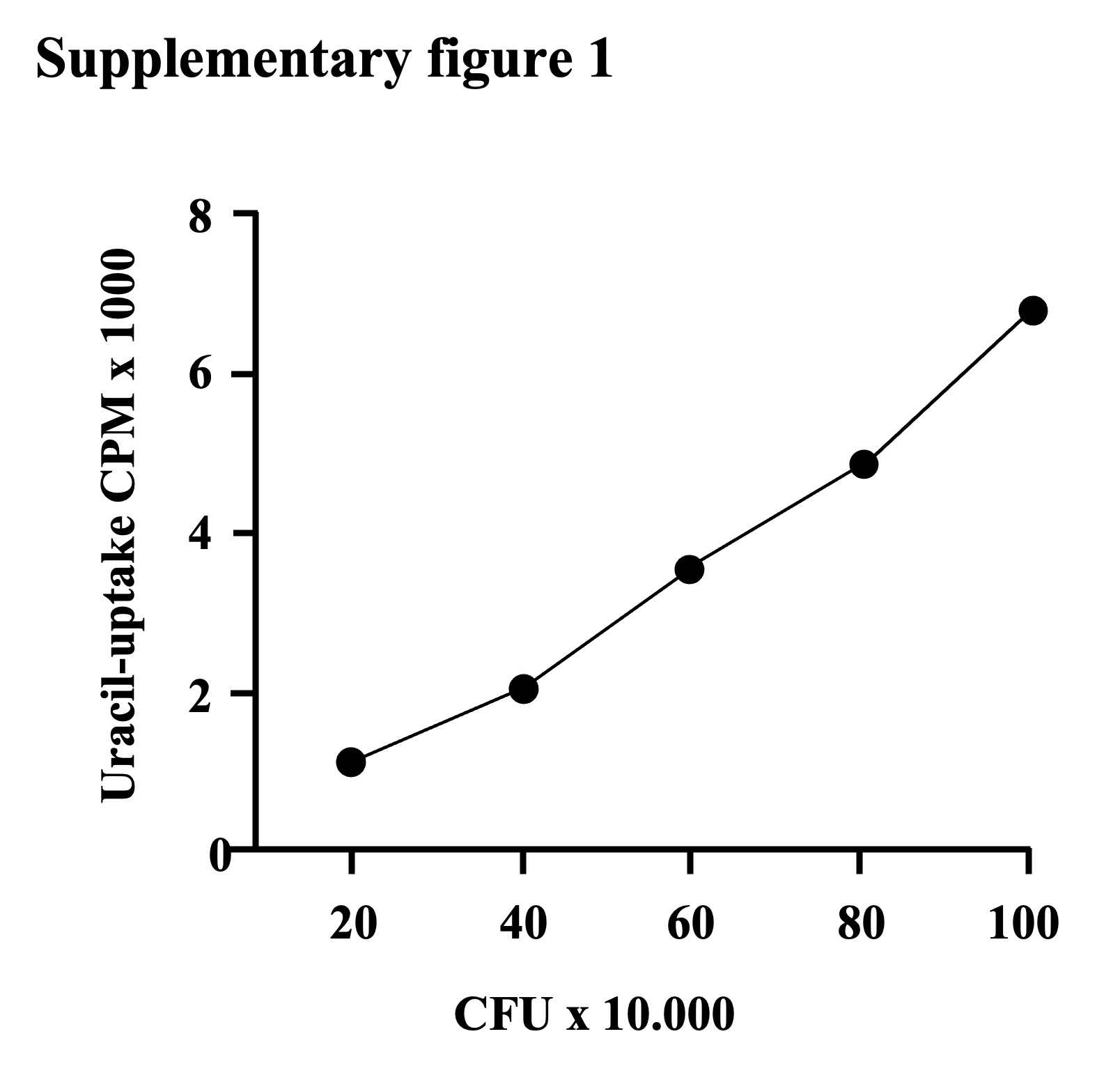

Supplement: SUPPLEMENTARY FIGURE 1 — Detection of HBB 112–147. Fragmentation spectrum showing the presence of HBB 112–147, VCVLAHHFGKEFTPPVQAAYQKVVAGVANALAHKYH. [file Image_1.JPEG]

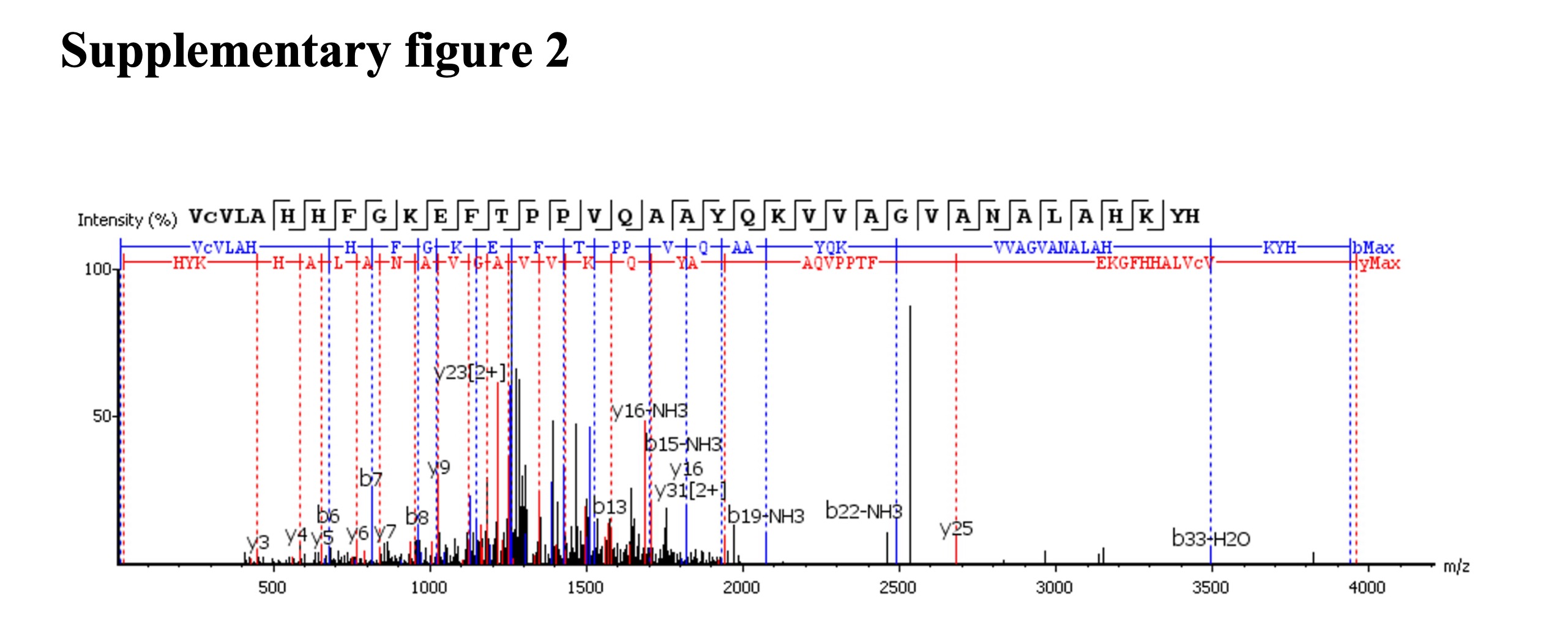

Supplement: SUPPLEMENTARY FIGURE 2 — Detection of RapTB with hemoglobin-ß antibody. After Immunofluorescence staining of RapTB using an anti-HBB antibody and a Cy2-labelled secondary antibody, fluorescent emission is measured. The figure shows a titration of RapTB: 50 μM (A), 5 μM (B), 0.5 μM (C) und 0.05 μM (D). [file Image_2.JPEG]

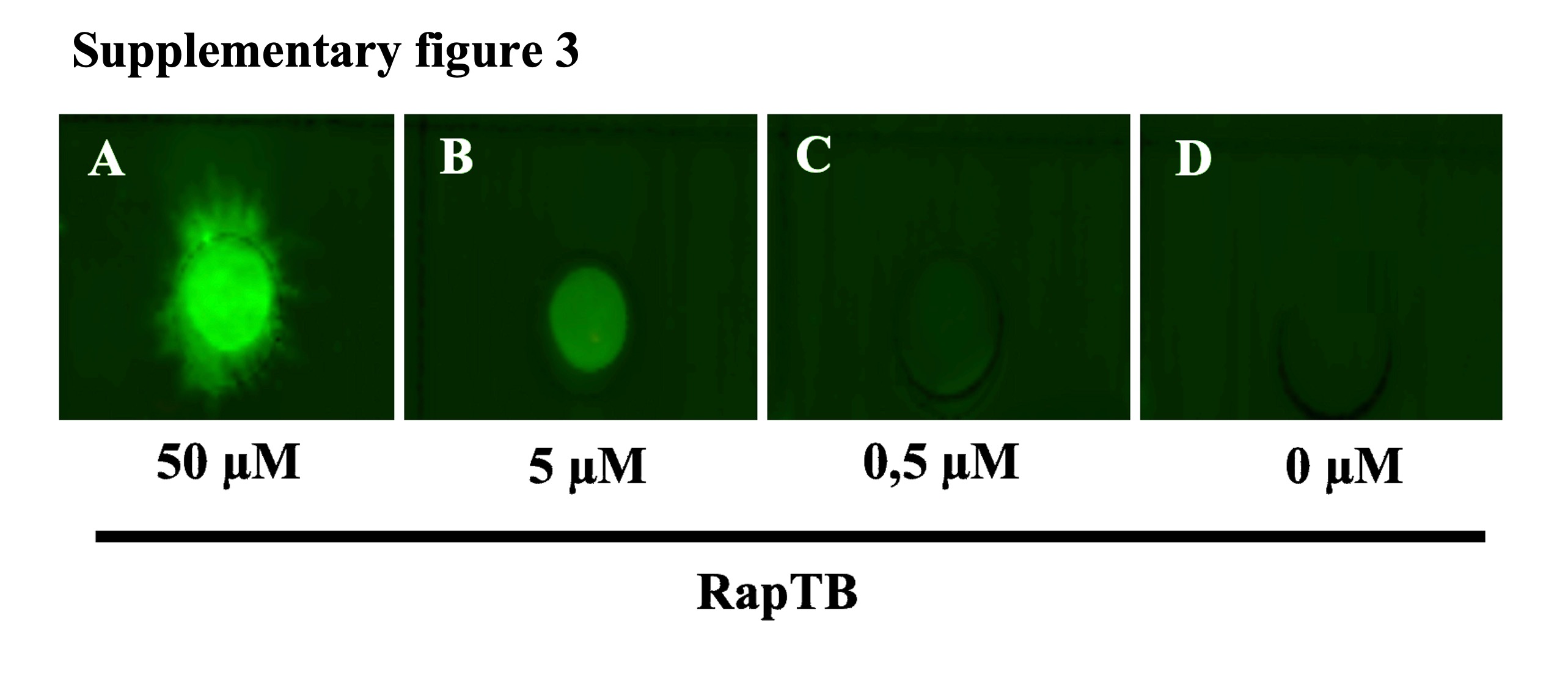

Supplement: Supplementary file 3 [file Image_3.JPEG]
